# Coverage with Influenza, Respiratory Syncytial Virus, and COVID-19 Vaccines Among Nursing Home Residents — National Healthcare Safety Network, United States, November 2024

**DOI:** 10.15585/mmwr.mm7346a2

**Published:** 2024-11-21

**Authors:** Hannah E. Reses, George Segovia, Heather Dubendris, Kira Barbre, Sushmitha Ananth, Brynn Lape-Newman, Emily Wong, Molly Stillions, Theresa Rowe, Elizabeth Mothershed, Erika Wallender, Evelyn Twentyman, Ryan E. Wiegand, Pragna Patel, Andrea Benin, Jeneita M. Bell

**Affiliations:** ^1^Division of Healthcare Quality Promotion, National Center for Emerging and Zoonotic Infectious Diseases, CDC; ^2^Chenega Enterprise, Systems & Solutions, LLC, Chesapeake, Virginia; ^3^Lantana Consulting Group, East Thetford, Vermont; ^4^Goldbelt C6, Chesapeake, Virginia; ^5^Leidos, Inc, Reston, Virginia; ^6^Immunization Services Division, National Center for Immunization and Respiratory Diseases, CDC; ^7^Coronavirus and Other Respiratory Viruses Division, National Center for Immunization and Respiratory Diseases, CDC.

**Summary**

**What is already known about this topic?**

Nursing home residents are at increased risk for severe COVID-19, influenza, and respiratory syncytial virus (RSV) illness. Vaccination reduces risk for severe outcomes from these vaccine-preventable respiratory diseases.

**What is added by this report?**

As of November 10, 2024, 30% of nursing home residents had received a 2024–2025 COVID-19 vaccine. Among residents at nursing home facilities that elected to report vaccination against influenza (59% of facilities) and RSV (52% of facilities), 58% had received influenza vaccination, and only 18% had received RSV vaccination.

**What are the implications for public health practice?**

Addressing low vaccination coverage against COVID-19, influenza, and RSV is a priority to protect residents susceptible to severe outcomes of respiratory illnesses.

## Abstract

Nursing home residents are at elevated risk for severe complications from respiratory viruses, including SARS-CoV-2, influenza, and respiratory syncytial virus (RSV). Nursing homes are required to report COVID-19 vaccination coverage and can voluntarily report influenza and RSV vaccination coverage among residents to CDC’s National Healthcare Safety Network. The purpose of this study was to assess COVID-19, influenza, and RSV vaccination coverage among nursing home residents early in the 2024–25 respiratory virus season. As of November 10, 2024, 29.7% of nursing home residents had received a 2024–2025 COVID-19 vaccine. Among residents at facilities that elected to report vaccination against influenza (59.4% of facilities) and RSV (51.8% of facilities), 58.4% had received influenza vaccination, and 17.9% had received RSV vaccination. Vaccination coverage varied by U.S. Department of Health and Human Services region, social vulnerability index level, and facility size. Addressing low coverage with COVID-19, influenza, and RSV vaccines is a priority to protect residents who are susceptible to severe outcomes associated with respiratory illnesses.

## Introduction

Nursing home residents are at elevated risk for severe complications from respiratory viruses, including SARS-CoV-2 (*1*), influenza (*2*), and respiratory syncytial virus (RSV) (*3*). The Advisory Committee on Immunization Practices (ACIP) recommends that all persons aged ≥6 months receive a 2024–2025 COVID-19 vaccine* and an annual influenza vaccine.^†^ ACIP also recommends that all nursing home residents aged ≥60 years receive 1 dose of RSV vaccine (*4*). Since 2021, the Centers for Medicare & Medicaid Services (CMS) has required nursing homes to report weekly aggregate COVID-19 vaccination coverage among residents to CDC’s National Healthcare Safety Network (NHSN).^§^ Since October 2023, nursing homes may also voluntarily report weekly aggregate resident influenza and RSV vaccination coverage data to NHSN.^¶^ The purpose of this study was to assess COVID-19, influenza, and RSV vaccination coverage among nursing home residents early in the 2024–25 respiratory virus season.

## Methods

### Data Collection and Analysis

Nursing homes electronically report the number of residents who occupied a bed at the facility for ≥1 day during the week of data collection and the number of residents who received the 2024–2025 COVID-19 and influenza vaccines and RSV vaccine. Data reported from CMS-certified nursing homes for the week of November 10, 2024, were used for analysis.** Representativeness of facilities voluntarily reporting influenza and RSV vaccination coverage was assessed by comparing facility and county characteristics among reporting facilities to all facilities enrolled in NHSN. Estimates of coverage (percentage of residents vaccinated) with COVID-19, influenza, and RSV vaccines, and 95% CIs were estimated using Poisson regression models. For each vaccine, coverage was stratified by U.S. Department of Health and Human Services (HHS) region,^††^ county-level social vulnerability index (SVI) tertile,^§§^ and facility-size tertile.^¶¶^ All analyses were conducted using SAS (version 9.4; SAS Institute). Nonoverlapping 95% CIs were considered to represent statistically significant differences. This activity was reviewed by CDC, deemed not research, and was conducted consistent with applicable federal law and CDC policy.***

## Results

### Representativeness of Voluntary Influenza and RSV Reporters

Influenza and RSV vaccination coverage as of November 10, 2024, was voluntarily reported by 8,974 (59.4%) and 7,816 (51.8%), respectively, among 15,100 CMS-certified nursing homes. Among facilities voluntarily reporting coverage, the distributions of facilities by HHS region, SVI, and facility size were comparable to distributions among all CMS-certified nursing homes enrolled in NHSN (Table).

**TABLE:** Estimates* of 2024–2025 COVID-19, annual influenza, and respiratory syncytial virus vaccination coverage among nursing home residents — National Healthcare Safety Network, United States, November 2024^†^

Characteristic	Total no. of facilities (%)	2024–2025 COVID-19 vaccination coverage				Influenza vaccination coverage				RSV vaccination coverage				
		No. of facilities reporting (%)	No. of residents	No. of vaccinated residents	Coverage, % (95% CI)	No. of facilities reporting (%)	No. of residents	No. of vaccinated residents	Coverage, % (95% CI)	No. of facilities reporting (%)		No. of residents	No. of vaccinated residents	Coverage, % (95% CI)
Total	15,100 (100)	14,028 (92.9)	1,218,546	361,869	29.7 (29.6–29.8)	8,974 (59.4)	765,333	447,108	58.4 (58.2–58.6)	7,816 (51.8)		661,075	118,433	17.9 (17.8–18.0)
**HHS Region^§^**														
Region 1	**817 (5.4)**	769 (5.5)	73,106	24,531	33.6 (33.1–34.0)	497 (5.5)	45,007	28,832	64.1 (63.3–64.8)	441 (5.6)		40,050	8,059	20.1 (19.7–20.6)
Region 2	**972 (6.4)**	923 (6.6)	137,894	40,691	29.5 (29.2–29.8)	655 (7.3)	94,823	57,277	60.4 (59.9–60.9)	570 (7.3)		81,664	16,139	19.8 (19.5–20.1)
Region 3	**1,385 (9.2)**	1,277 (9.1)	130,990	39,563	30.2 (29.9–30.5)	762 (8.5)	76,113	47,501	62.4 (61.8–63.0)	674 (8.6)		66,698	12,815	19.2 (18.9–19.5)
Region 4	**2,686 (17.8)**	2,542 (18.1)	243,111	59,869	24.6 (24.4–24.8)	1,706 (19.0)	163,452	87,206	53.4 (53.0–53.7)	1,425 (18.2)		133,244	16,080	12.1 (11.9–12.3)
Region 5	**3,278 (21.7)**	2,940 (21.0)	226,122	70,236	31.1 (30.8–31.3)	1,841 (20.5)	136,077	78,153	57.4 (57.0–57.8)	1,659 (21.2)		121,830	26,265	21.6 (21.3–21.8)
Region 6	**2,056 (13.6)**	1,934 (13.8)	147,304	29,175	19.8 (19.6–20.0)	1,114 (12.4)	83,897	49,958	59.5 (59.0–60.1)	908 (11.6)		68,727	6,405	9.3 (9.1–9.6)
Region 7	**1,426 (9.4)**	1,312 (9.4)	79,373	29,140	36.7 (36.3–37.1)	848 (9.4)	50,183	30,769	61.3 (60.6–62.0)	752 (9.6)		44,303	9,922	22.4 (22.0–22.8)
Region 8	**585 (3.9)**	550 (3.9)	34,996	13,520	38.6 (38.0–39.3)	347 (3.9)	20,722	13,119	63.3 (62.2–64.4)	319 (4.1)		19,312	5,642	29.2 (28.5–30.0)
Region 9	**1,471 (9.7)**	1,376 (9.8)	119,747	46,026	38.4 (38.1–38.8)	951 (10.6)	78,819	46,025	58.4 (57.9–58.9)	842 (10.8)		70,468	14,044	19.9 (19.6–20.3)
Region 10	**424 (2.8)**	405 (2.9)	25,903	9,118	35.2 (34.5–35.9)	253 (2.8)	16,240	8,268	50.9 (49.8–52.0)	226 (2.9)		14,779	3,062	20.7 (20.0–21.5)
**SVI^¶^**														
Low	**5,027 (33.4)**	4,658 (33.2)	368,111	123,812	33.6 (33.4–33.8)	2,995 (33.4)	230,535	140,221	60.8 (60.5–61.1)	2,697 (34.5)		206,461	43,950	21.3 (21.1–21.5)
Medium	**5,037 (33.4)**	4,671 (33.3)	430,902	122,844	28.5 (28.3–28.7)	2,904 (32.4)	263,658	150,058	56.9 (56.6–57.2)	2,492 (31.9)		222,922	38,968	17.5 (17.3–17.7)
High	**5,006 (33.2)**	4,672 (33.3)	417,151	114,261	27.4 (27.2–27.6)	3,051 (34.0)	268,848	155,162	57.7 (57.4–58.0)	2,609 (33.4)		230,245	35,247	15.3 (15.1–15.5)
Facility size**														
Small	**5,181 (34.3)**	4,691 (33.4)	199,347	69,131	34.7 (34.4–34.9)	3,170 (35.3)	133,628	84,094	62.9 (62.5–63.4)	2,778 (35.5)	117,250		28,242	24.1 (23.8–24.4)
Medium	**5,012 (33.2)**	4,682 (33.4)	368,525	110,595	30.0 (29.8–30.2)	2,955 (32.9)	232,199	136,676	58.9 (58.6–59.2)	2,583 (33.0)	203,116		35,932	17.7 (17.5–17.9)
Large	**4,907 (32.5)**	4,655 (33.2)	650,674	182,143	28.0 (27.9–28.1)	2,849 (31.7)	399,506	226,338	56.7 (56.4–56.9)	2,455 (31.4)	340,709		54,259	15.9 (15.8–16.1)

**Abbreviations**: HHS = U.S. Department of Health and Human Services; RSV = respiratory syncytial virus; SVI = social vulnerability index.

* Estimates of coverage (percentage of residents vaccinated) with COVID-19, influenza, and RSV vaccines, and 95% CIs were calculated using Poisson regression models.

^†^ Data reported from nursing homes for the week of November 10, 2024 (or the preceding week if data for November 10, 2024, were not available) were used for analysis. Facilities were excluded from the coverage estimates if they reported zero residents or did not report data for either the week of November 10, 2024, or the week of November 3, 2024 (1,072 facilities were excluded for COVID-19; 6,126 were excluded for influenza; and 7,284 were excluded for RSV).

^§^ https://www.hhs.gov/about/agencies/iea/regional-offices/index.html

^¶^ https://www.atsdr.cdc.gov/place-health/php/svi/?CDC_AAref_Val=https://www.atsdr.cdc.gov/placeandhealth/svi/index.html. SVI categories were based on the tertile distribution of the SVI of the counties where the facilities are located. Low: ≤0.38; medium: >0.38 to ≤0.66; and high: >0.66. Thirty facilities were located in counties that did not have an assigned SVI score.

** Facility size category was based on the tertile distribution of the total number of residents per facility. Small: ≤60 residents; medium: 61–96 residents; and large: ≥97 residents.

### 2024–2025 COVID-19 Vaccination Coverage

COVID-19 vaccination coverage was reported by 14,028 (92.9%) nursing homes. Approximately three in 10 (29.7%) nursing home residents received the 2024–2025 COVID-19 vaccine, with coverage ranging from 19.8% in HHS Region 6 to 38.6% in HHS Region 8. COVID-19 vaccination coverage was highest in the least socially vulnerable counties (33.6%) and in small facilities (34.7%) and lowest in large facilities (28.0%).

### Influenza Vaccination Coverage

Among the 59.4% of facilities that voluntarily reported influenza vaccination data, more than one half (58.4%) of residents had received an influenza vaccine. Coverage ranged from 50.9% in HHS Region 10 to 64.1% in HHS Region 1. Influenza vaccination coverage was highest in the least socially vulnerable counties (60.8%) and in small facilities (62.9%) and was lowest in large facilities (56.7%).

### RSV Vaccination Coverage

Among the 51.8% of nursing homes that voluntarily reported RSV vaccination data, approximately one in six (17.9%) residents had received an RSV vaccine. Coverage ranged from 9.3% in HHS Region 6 to 29.2% in HHS Region 8. RSV vaccination coverage was highest in the least socially vulnerable counties (21.3%) and in small facilities (24.1%) and lowest in the most socially vulnerable counties (15.3%) and large facilities (15.9%).

### Overall Vaccination Coverage by Region

Among HHS regions, HHS Region 8 (Colorado, Montana, North Dakota, South Dakota, Utah, and Wyoming) had the highest coverage with COVID-19 and RSV vaccines and the second highest coverage with influenza vaccine. Within Region 8, coverage with COVID-19 and RSV vaccines was highest in South Dakota, and influenza vaccination coverage was highest in North Dakota (Figure).

**FIGURE:**
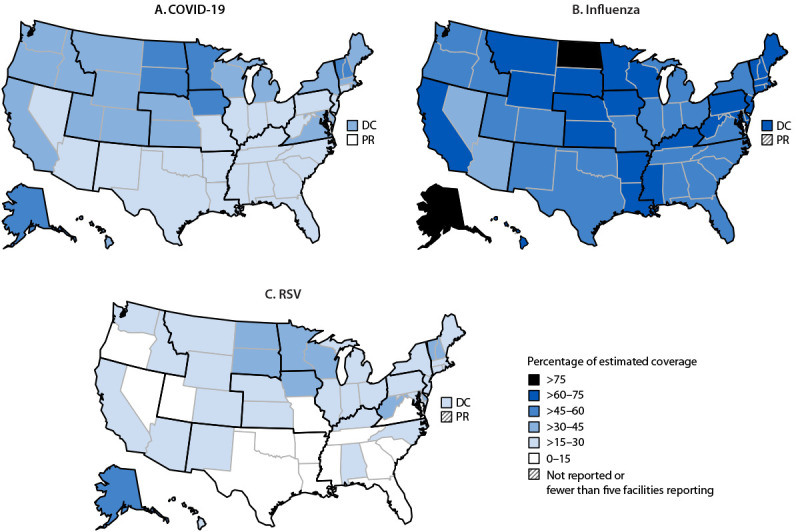
Estimates* of percentage of coverage with 2024–2025 COVID-19 vaccine (A), influenza vaccine (B), and respiratory syncytial virus vaccine (C) among nursing home residents, by U.S. jurisdiction and U.S. Department of Health and Human Services region^†^ — National Healthcare Safety Network, United States, November 2024^§^ **Abbreviations**: DC = District of Columbia; PR = Puerto Rico; RSV = respiratory syncytial virus. * Estimates of coverage (percentage of residents vaccinated) with 2024–2025 COVID-19, influenza, and RSV vaccine and 95% CIs were calculated using Poisson regression models. ^†^ https://www.hhs.gov/about/agencies/iea/regional-offices/index.html ^§^ Data reported from nursing homes for the week of November 10, 2024 (or the preceding week if data for November 10, 2024, were not available) were used for analysis. Facilities were excluded from the coverage estimates if they reported zero residents or did not report data for either the week of November 10, 2024, or the week of November 3, 2024 (1,072 facilities were excluded for COVID-19; 6,126 were excluded for influenza; and 7,284 were excluded for RSV).

## Discussion

As of November 10, 2024, coverage with COVID-19, influenza, and RSV vaccines among nursing home residents reported to NHSN was low. Compared with coverage among nursing home residents reported to NHSN as of November 12, 2023, this season’s COVID-19 vaccination coverage was higher (29.7% versus 24.0% in 2023),^†††^ but influenza vaccination coverage was lower (58.4% versus 68.3% in 2023) (NHSN, unpublished data, 2023). Coverage with a one-time dose of RSV vaccine increased from 6.7% as of November 12, 2023, to 17.9% as of November 10, 2024 (NHSN, unpublished data 2023). Compared with COVID-19 vaccination coverage among adults aged ≥65 years and RSV vaccination coverage among adults aged ≥75 years reported by the National Immunization Survey (NIS) Adult COVID-19 Module for the week ending November 9, 2024, (38.5% and 39.7%, respectively), COVID-19 and RSV vaccination coverage among nursing home residents reported to NHSN was lower (29.7% and 17.9%, respectively). In contrast, influenza vaccination coverage among nursing home residents (58.4%) was similar to that of the general adult population aged ≥65 years (58.6%) (*5*). Although data from NHSN and NIS cannot be directly compared because of methodological differences, these directional differences in COVID-19 and RSV vaccination coverage are consistent with those observed during the 2023–24 respiratory virus season (*6*) and might reflect barriers to respiratory virus vaccination in nursing homes.

During each week of October 2024, approximately 5,500 nursing home residents received a diagnosis of COVID-19,^§§§^ and 360 were hospitalized after having received a positive COVID-19 test result (NHSN, unpublished data, 2024).^¶¶¶^ During the 2023–24 respiratory virus season, adults aged ≥65 years accounted for 70% of all COVID-19–associated hospitalizations, and approximately one in six adults hospitalized with COVID-19 was a nursing home resident (*7*). These findings are consistent with a report indicating that COVID-19–associated hospitalizations among nursing home residents during the 2023–24 respiratory virus season peaked at 7.1 per 10,000 residents, approximately eight times the peak weekly rate of 0.87 per 10,000 among all U.S. adults aged ≥70 years, the age group with the highest COVID-19–associated hospitalization rate (*8*).

Demand for vaccines is low among nursing home residents, as it is among other U.S. populations.**** Mistrust in institutions and concerns about safety, efficacy, the rapid development and approval of the vaccines, and type of vaccine (i.e., mRNA) are the most commonly reported factors contributing to COVID-19 vaccine hesitancy (*9*). Misinformation and doubts regarding efficacy also might contribute to hesitancy about other vaccines (*9*). A strong recommendation for vaccination by facility health care providers and leadership can help overcome vaccine hesitancy among residents and their families.^††††^ During both the 2023–24 respiratory virus season and the 2024–25 season to date, coverage with all three vaccines was highest in small nursing homes and in nursing homes in North Dakota and South Dakota, suggesting that staff members in small facilities might be better able to build trust with residents and families and mitigate barriers to vaccination (*6*) and that efforts by states to develop strong relationships among stakeholders are effective.^§§§§^

Among persons aged ≥60 years hospitalized with RSV during July 2022–June 2023, 17% were long-term care residents (*3*); in June 2024, ACIP expanded its recommendation for RSV vaccine to include vaccination of all nursing home residents aged ≥60 years (*4*). However, RSV vaccination coverage among nursing home residents remains low, suggesting that barriers to vaccination still exist. The higher cost of RSV^¶¶¶¶^ and COVID-19***** vaccines, compared with influenza vaccine, can increase the cost of vaccination for nursing homes and residents. RSV vaccines are billable to Medicare Part D, and some nursing homes do not have systems in place for Part D billing. This study found that coverage with all three vaccines was highest in counties with lower social vulnerability, suggesting that barriers related to cost and billing disproportionately affect nursing homes with limited resources.

Given the multiple barriers to achieving high vaccination coverage, a combination of approaches is needed. CDC and CMS developed the Billing Medicare for Respiratory Vaccines fact sheet for nursing homes.^†††††^ CDC provides NHSN surveillance data to state and local health departments and CMS Quality Innovation Networks-Quality Improvement Organizations to help guide direct outreach to facilities with low COVID-19 vaccination coverage. CDC has also increased its tailored guidance and supporting materials for nursing home settings and older adults, including webinars, the Viral Respiratory Pathogens Toolkit for Nursing Homes, and the Why Get Vaccinated counseling sheet.^§§§§§^ In addition, the HHS-funded Risk Less. Do More. campaign to improve vaccine confidence is aimed at older adults and includes a focus on nursing home residents.^¶¶¶¶¶^

### Limitations

The findings of this report are subject to at least three limitations. First, reporting of influenza and RSV vaccination coverage is optional. Facilities that elected to report these data might be more likely to offer influenza or RSV vaccines. However, similarities in distribution of facility characteristics between voluntary reporters and all facilities suggest that facilities voluntarily reporting these data are similar to all facilities. Second, a substantial increase in the number of facilities reporting influenza vaccination (from 22.1% to 59.4%) and RSV vaccination (from 21.1% to 51.8%) between the previous season (the week of November 12, 2023) and this season (the week of November 10, 2024), limits direct comparison. Finally, vaccination data reported to NHSN are not disaggregated by age; RSV vaccination coverage was calculated among residents of all ages, not just the approximately 91% of nursing home residents aged ≥60 years (*10*). RSV vaccination coverage among residents aged ≥60 years was likely higher than the overall reported coverage.

### Implications for Public Health Practice

Most nursing home residents have not been afforded the protection offered by vaccination against severe COVID-19, influenza, and RSV disease during the 2024–25 respiratory virus season. Addressing low coverage of vaccination against COVID-19, influenza, and RSV must be prioritized, and larger facilities and those in counties with high social vulnerability could benefit from effective interventions. Although CDC and other federal agencies have programs in place to address both the financial and vaccine hesitancy–related barriers to vaccination in nursing homes, more needs to be done at every level to protect nursing home residents, who constitute one of the population groups at highest risk for severe respiratory disease.

## Footnotes

* As of October 23, 2024, ACIP also recommends that persons aged ≥65 years and those who are moderately or severely immunocompromised receive a second dose of 2024–2025 COVID-19 vaccine 6 months after receipt of the first dose (https://www.cdc.gov/covid/vaccines/stay-up-to-date.html). The data used for this study were collected <6 months after the 2024–2025 COVID-19 vaccine was released and reflect receipt of the first dose of 2024–2025 COVID-19 vaccine.

† https://www.cdc.gov/flu/vaccines/index.html

§
https://www.federalregister.gov/d/2021-10122

¶
https://www.cdc.gov/nhsn/pdfs/covid19/ltcf/vax-rpv-protocol-ltc-residents-oct-2023-508.pdf

** Facilities were included if they were actively enrolled in the NHSN Long-Term Care Component and had ever reported nonzero vaccination denominator data. Facilities were excluded from the coverage estimates if they reported zero residents or did not report data for either the week of November 10, 2024, or the week of November 3, 2024 (1,072 facilities were excluded for COVID-19; 6,126 were excluded for influenza; and 7,284 were excluded for RSV).

††
https://www.hhs.gov/about/agencies/iea/regional-offices/index.html

§§ SVI considers socioeconomic status, household characteristics, race, ethnicity, housing type, and transportation. Counties in a lower SVI tertile are less socially vulnerable than are those in an upper SVI tertile. SVI categories were based on the tertile distribution of the SVI of the counties where the facilities are located (low: ≤0.38; medium: >0.38 to ≤0.66; and high: >0.66). Thirty facilities were excluded because they were located in counties that did not have an assigned SVI score. https://www.atsdr.cdc.gov/place-health/php/svi/?CDC_AAref_Val=https://www.atsdr.cdc.gov/placeandhealth/svi/index.html

¶¶ Facility size category was based on the tertile distribution of the total number of residents per facility (small: ≤60 residents; medium: 61–96 residents; and large: ≥97 residents).

*** 45 C.F.R. part 46.102(l)(2), 21 C.F.R. part 56; 42 U.S.C. Sect. 241(d); 5 U.S.C. Sect. 552a; 44 U.S.C. Sect. 3501 et seq.

†††
https://www.cdc.gov/nhsn/covid19/ltc-vaccination-dashboard.html

§§§
https://www.cdc.gov/nhsn/covid19/ltc-report-overview.html

¶¶¶ NHSN defines COVID-19–associated hospitalization as a hospital admission within 10 days after a laboratory-confirmed SARS-CoV-2 infection.

****
https://www.cdc.gov/covidvaxview/weekly-dashboard/adult-vaccination-coverage.html

††††
https://www.cdc.gov/long-term-care-facilities/hcp/respiratory-virus-toolkit/index.html

§§§§ https://www.washingtonpost.com/opinions/2023/12/05/covid-vaccine-nursing-homes-dakota/; https://www.washingtonpost.com/opinions/2023/12/07/lessons-nursing-home-covid-vaccine/?utm_campaign

¶¶¶¶
https://www.mcknights.com/news/ask-the-care-expert-about-billing-for-rsv-vaccines/

*****
https://www.mcknights.com/news/new-covid-vaccines-available-but-payment-issues-complicate-access-for-nursing-homes/

†††††
https://www.cms.gov/files/document/billing-medicare-respiratory-vaccines.pdf

§§§§§
https://www.cdc.gov/vaccines/hcp/downloads/ltcf-why-get-vaccinated.pdf

¶¶¶¶¶
https://www.hhs.gov/risk-less-do-more/index.html

## References

[1] Bagchi S, Mak J, Li Q, Rates of COVID-19 among residents and staff members in nursing homes—United States, May 25–November 22, 2020. MMWR Morb Mortal Wkly Rep 2021;70:52–5. 10.15585/mmwr.mm7002e2 33444301 PMC7808710

[2] Lansbury LE, Brown CS, Nguyen-Van-Tam JS. Influenza in long-term care facilities. Influenza Other Respir Viruses 2017;11:356–66. 10.1111/irv.12464 28691237 PMC5596516

[3] Havers FP, Whitaker M, Melgar M, Characteristics and outcomes among adults aged ≥60 years hospitalized with laboratory-confirmed respiratory syncytial virus—RSV-NET, 12 states, July 2022–June 2023. MMWR Morb Mortal Wkly Rep 2023;72:1075–82. 10.15585/mmwr.mm7240a1 37796742 PMC10564327

[4] Britton A, Roper LE, Kotton CN, Use of respiratory syncytial virus vaccines in adults aged ≥60 years: updated recommendations of the Advisory Committee on Immunization Practices—United States, 2024. MMWR Morb Mortal Wkly Rep 2024;73:696–702. 10.15585/mmwr.mm7332e1 39146277

[5] Kriss JL, Black CL, Razzaghi H, Influenza, COVID-19, and respiratory syncytial virus vaccination coverage among adults—United States, fall 2024. MMWR Morb Mortal Wkly Rep 2024;73:1044–57. https://www.cdc.gov/mmwr/volumes/73/wr/mm7346a1.htm?s_cid=mm7346a1_w 10.15585/mmwr.mm7346a1 PMC11581203 39570786

[6] Reses HE, Dubendris H, Haas L, Coverage with influenza, respiratory syncytial virus, and updated COVID-19 vaccines among nursing home residents—National Healthcare Safety Network, United States, December 2023. MMWR Morb Mortal Wkly Rep 2023;72:1371–6. 10.15585/mmwr.mm7251a3 38127673 PMC10754267

[7] Taylor CA, Patel K, Pham H, COVID-19–associated hospitalizations among U.S. adults aged ≥18 years—COVID-NET, 12 states, October 2023–April 2024. MMWR Morb Mortal Wkly Rep 2024;73:869–75. 10.15585/mmwr.mm7339a2 39361542 PMC11449267

[8] Franklin D, Barbre K, Rowe TA, COVID-19 vaccination coverage, and rates of SARS-CoV-2 infection and COVID-19–associated hospitalization among residents in nursing homes—National Healthcare Safety Network, United States, October 2023–February 2024. MMWR Morb Mortal Wkly Rep 2024;73:339–44. 10.15585/mmwr.mm7315a3 38635474 PMC11037435

[9] Nwachukwu G, Rihan A, Nwachukwu E, Uduma N, Elliott KS, Tiruneh YM. Understanding COVID-19 vaccine hesitancy in the United States: a systematic review. Vaccines (Basel) 2024;12:747. 10.3390/vaccines12070747 39066385 PMC11281578

[10] Tu W, Li R, Stump TE, Age-specific rates of hospital transfers in long-stay nursing home residents. Age Ageing 2022;51:afab232. 10.1093/ageing/afab232 34850811

